# GENEOnet: a breakthrough in protein binding pocket detection using group equivariant non-expansive operators

**DOI:** 10.1038/s41598-025-18132-5

**Published:** 2025-10-03

**Authors:** Giovanni Bocchi, Patrizio Frosini, Alessandra Micheletti, Alessandro Pedretti, Gianluca Palermo, Davide Gadioli, Carmen Gratteri, Filippo Lunghini, Akash Deep Biswas, Pieter F. W. Stouten, Andrea R. Beccari, Anna Fava, Carmine Talarico

**Affiliations:** 1 Department of Environmental Science and Policy, Università degli Studi di Milano, Via Celoria 10, 20133 Milano, Italy; 2 Department of Computer Science, University of Pisa, Largo B. Pontecorvo 3, 56127 Pisa, Italy; 3 Department of Pharmaceutical Sciences, Università degli Studi di Milano, Via Mangiagalli 25, 20133 Milano, Italy; 4 Department of Electronics, Information and Bioengineering, Politecnico di Milano, Via Ponzio 34/5, 20133 Milano, Italy; 5 LIGHT S.c.a.r.l., Via Branze 45, 25123 Brescia, Italy; 6 Dompé Farmaceutici S.p.A., Via Tommaso de Amicis 95, 80145 Napoli, Italy; 7 Stouten Pharma Consultancy BV, Kempenarestraat 47, 2860 Sint-Katelijne-Waver, Belgium

**Keywords:** Equivariance, GENEO, Molecular docking, Pocket detection, Cheminformatics, Kinases, Machine learning, Protein analysis, Applied mathematics, Statistics

## Abstract

Structure-based virtual screening approaches like molecular docking rely on accurately identifying and precisely calculating binding pockets to efficiently search for potential ligands. In this paper, we introduce GENEOnet, a machine learning model designed for volumetric protein pocket detection that employs Group Equivariant Non-Expansive Operators (GENEOs). These operators simplify model complexity and enable more informed domain knowledge integration by selecting specific physical and chemical properties for each operator to focus on, as well as how they should react. Unlike other methods in this field, GENEOnet has fewer model parameters, resulting in reduced training costs, and offers greater explainability, allowing the parameters to be easily interpreted. GENEOnet processes the empty space within a protein by converting it into a 3D grid of uniform blocks, known as ‘voxels’. It then identifies regions of the grid with an output value above a threshold, thus producing a list of predicted pockets, ranked according to the model’s average output value. Our experimental results show that GENEOnet performs robustly even with small training datasets of 200 proteins and surpasses other established state-of-the-art methods in various metrics. Specifically, GENEOnet’s $H_1$ score indicating the probability that the top-ranked pocket is the correct one is 0.764, compared to 0.702 for P2Rank, the next best performing algorithm on our PDBbind test set. Moreover, a case study considering various ABL1 kinase conformations demonstrates the excellent agreement between GENEOnet’s predictions and experimental sites. GENEOnet is available as a web service at https://geneonet.exscalate.eu, where users can access the pre-trained model for detecting and ranking protein cavities.

## Introduction

### Introduction to Exscalate platform

The field of drug discovery is undergoing a significant transformation with advancements in computational methods. The Exscalate platform, developed by Dompé, is at the forefront of this evolution. This high-throughput system allows for virtual screening and drug design, offering a faster and more cost-effective alternative to traditional experimental approaches. Exscalate’s strength lies in its ability to integrate multiple aspects of computational chemistry and biology, such as binding site generation, docking, toxicity prediction, and cheminformatics. By leveraging a unique chemical cartridge, the platform can process vast chemical libraries, analyzing trillions of compounds to identify potential drug candidates with precision^1–3^. A critical aspect of Exscalate is its capability to detect and analyze protein binding pockets regions on proteins where ligands can form stable interactions. Accurate identification of these sites is crucial in understanding how ligands bind, as their properties significantly impact the affinity and specificity of the interaction. As show in this paper, through the integration of machine learning and sophisticated molecular modeling, Exscalate can identify these critical sites even without clear experimental evidence. The platform’s efficiency is further demonstrated by LiGen, a tool for ultra-high-throughput docking that can process tens of trillions of compounds using advanced GPU technology. This capability showcases the robust computational power of the Exscalate platform and Dompé’s commitment to global health initiatives, such as the pro bono discovery of inhibitors for Covid-19 and Zika viruses^4–6^. The integration of these sophisticated computational techniques makes the Exscalate platform a significant advancement in drug design, promising to expedite the discovery of new therapeutics while reducing costs and broadening access to innovative treatments across the global healthcare landscape.

### Introduction to protein pocket detection

The growing capabilities of computational technology have increasingly enabled in silico techniques to play a significant role in drug discovery and repositioning processes^7^. Various computational approaches^8^, such as structure-based drug design^9^, have emerged as effective tools for predicting binding affinity, identifying interacting substructures^10,11^, and understanding the mechanism of action of pharmacologically active agents^12^. A precise characterization of the binding pocket is essential for efficient docking calculations^13^. Blind docking analyses that involve the entire target protein tend to be less effective in finding correct ligand poses^14^. To overcome this challenge, researchers often rely on co-crystallized ligand-receptor complexes or site-directed mutagenesis studies^15,16^ to identify binding sites. However, when such data are not available, accurately characterizing the binding site becomes more difficult, even with an experimentally resolved protein structure^17^. In these cases, identifying allosteric/accessory binding sites and assessing their druggability is crucial for optimizing virtual screening campaigns. To address this challenge, many algorithms have been developed to identify and characterize putative binding sites. These tools involve both geometric analysis of empty regions within a protein structure and physicochemical analysis to prioritize and identify the correct binding site(s). Geometric detection methods can be categorized into grid-based and grid-free approaches. Grid-based methods include algorithms that analyze grids of voxels (i.e. the 3D equivalent of pixels) to determine which points are within the protein structure and meet specific geometric and physicochemical requirements. Examples of grid-based methods include POCKET^18^, which scans a grid to detect patterns of protein-solvent-protein points in order to locate empty regions surrounded by protein atoms. CAVIAR^19^ further enhances this method by computing descriptors to predict binding site “ligandability”. CavVis^20^, instead, exploits volumes to detect cavities in combination with an analysis of Gaussian surfaces to approximate the solvent-excluded surface. In contrast, grid-free approaches use spherical probes placed on the protein surface and clustered according to representative properties of candidate pockets. Other grid-free methods, such as Fpocket^21^, use alpha spheres to detect local curvatures on the protein surface. Due to recent computational advancements, machine learning and deep learning techniques are now being applied to binding site prioritization. P2Rank^22^ uses random forests to evaluate the capability of each surface point to bind a ligand. DeepSite^23^ and DeepPocket^24^, instead, employ Convolutional Neural Networks (CNNs).

### Introduction to group equivariant non-expansive operators

Recent advancements in deep learning have highlighted the significance of equivariant operators in enhancing transparency, interpretability, and simplifying the training phase of neural networks^25–33^. Group Equivariant Non-Expansive Operators (GENEOs) are closely related to the concept of eXplainable Artificial Intelligence (XAI), which aims to develop methods that can be understood and trusted by humans^34,35^. GENEOs have been introduced as elementary components to build new types of networks (for mathematical definitions and more details refer to^36–38^) by exploiting the possibility of combining different operators using suitable operations. Operators that process data are often compatible with specific geometric transformations, a property called equivariance. Image blurring is an example of such an operation, for example we can say that image blurring is equivariant with respect to rotations: we obtain the same result if we first blur an image and then rotate it as if we first rotate it and then blur it. Operators often possess additional properties, such as non-expansivity. Non-expansivity, among other benefits, guarantees stability to small perturbations of the input data. For example, if one blurs an image slightly corrupted by white noise, the result should not be very different from the blurring of the uncorrupted image. GENEOs networks combine equivariance and non-expansivity and allows for a kind of “geometric knowledge engineering” of such models enhancing transparency by integrating information into operators that process data while also reducing parameters involved with respect to non-equivariant models.

### Structure of the paper

This paper presents an investigation into the application of the GENEO model, specifically GENEOnet, to identify protein pockets. The first section describes the sources of data used in this study (Section “Data sources”). We then introduce GENEOnet (Section “The GENEOnet model”), a network model that leverages empirical knowledge and exploits the equivariance properties of GENEOs to detect protein pockets. The specific problem of pocket detection is well-suited for GENEO-based approaches, as it relies on relevant empirical knowledge regarding binding site preference for lipophilic areas and hydrogen bonding opportunities. Moreover, pocket detection is equivariant to rotations and translations of the space, meaning that rotating or translating a protein does not alter its pockets but only their spatial orientation. Consequently, we define the GENEOnet network to be equivariant with respect to these transformations. The subsequent sections of this paper detail our methodology for training and selecting the GENEOnet model (Sections “Training”–“Model selection”) as well as comparing its performance with state-of-the-art methods (Section “Results”). We evaluate GENEOnet’s efficacy in both pocket identification (Section “Pocket identification and ranking”) volumetric goodness (Section “Overlap analysis”), distance metrics (Section “Distance metrics”), discussing the effects of equivariance and non-expansivity on its performance (Section “Equivariance and non-expansivity combined effect”), also by means of an ablation study (Section “Ablation study”). Moreover, we discuss an experiment regarding the computational times of the various methods (Section “Computational time evaluation”). Finally, a case study concerning kinases (Section “Structural analysis of ABL1 Kinase using GENEOnet”) highlights the coherence of GENEOnet predictions with experimental data. As additional validation, we compare GENEOnet with two other deep learning and generative AI methods on a representative example (Section “GENEOnet, TankBind and AF2BIND comparison on ABL1 Kinase”). Notably, Section “Webservice” presents the GENEOnet webservice. In addition to its technical merits, we highlight several advantages of GENEOnet, including a simpler model structure, fewer unknown parameters, and reduced data requirements for training. Notably, despite its relative simplicity, GENEOnet achieves superior results compared to methods relying on tens of thousands of trainable parameters. Finally, Section “Discussion and conclusions” presents our conclusions and provides a discussion of the implications of this work.

## Materials and methods

This section introduces the mathematical model that we use to identify pockets. We also explain how the model was trained together with the datasets used for training, model selection and testing.

### Data sources

During the training, validation, and testing of GENEOnet, two data sources were utilized:

- The PDBbind v.2020 database^39^. The PDBbind database provides binding affinity data for protein-ligand complexes deposited in the RCSB PDB^40^. The aim of using the PDBbind database is to provide high-quality datasets for drug design methods. A total of 12,295 protein-ligand complexes were retrieved, we will refer to this set as BIND. This set, as explained in the following, is subdivided into three parts:
  - TRAIN, which consists of 200 complexes sampled uniformly at random from the whole BIND. During the model selection phase, 200 sets of size 200 are considered as different TRAINs, eventually choosing the optimal one for deployment. To avoid reserving only part of BIND for sampling TRAIN proteins, we decided to sample them from the entire BIND dataset, this choice generates small intersections between each TRAIN and the sets defined in the next points.
  - BINDVAL, which consists of 3073 complexes (approximately 25% of the whole BIND), is used for model selection. Each of the TRAIN sets considered during model selection has an average intersection of 50 proteins with BINDVAL. In particular, the TRAIN version that is chosen as optimal has an intersection of 48 proteins with BINDVAL.
  - BINDTEST, which initially consists of 9222 complexes (approximately 75% of the whole BIND), is used for a first comparison of the model with other tools from the state-of-the-art. After training and model selection, the 152 molecules contained in the intersection between the chosen TRAIN and BINDTEST are removed. The final BINDTEST is made of 9070 complexes, and it is disjoint from both BINDVAL and the chosen TRAIN.
- The RCSB PDB is the largest resource for experimentally determined biomolecular structures, releasing new data daily. From this data source, 41,519 complexes were retrieved. First of all, we removed all the complexes already contained in BINDTEST, moreover, we also removed all the complexes whose ligands are classified as post-translational modifications (PTMs), which are of small interest from a pharmaceutical perspective. After this, we obtained a set of 33,341 complexes that we will denote as BANK, which is completely disjoint from BINDTEST.

Furthermore, to the sets that have been used for comparisons with other models, i.e., BINDTEST and BANK, an additional preprocessing step was applied: all the proteins having sequence identity larger than 80% with any of the proteins in the chosen TRAIN, after the model selection phase, were discarded. This was done to avoid possible bias in favour of GENEOnet and to guarantee a fair comparison with the other methods. Finally, after this step, BINDTEST contains 6854 proteins and BANK 28382.

**Fig. 1:**
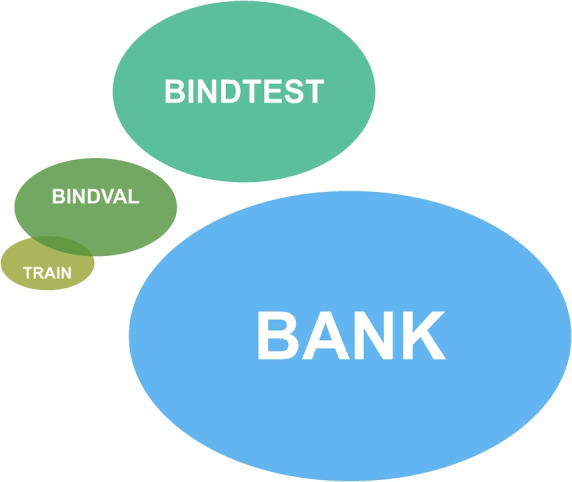
Datasets. Visual representation of the different datasets that will be considered. The chosen TRAIN set after model selection has an intersection of 48 proteins with BINDVAL, while BINDTEST, which is made of 6854 complexes, is completely disjoint from both of them. BANK is the largest set consisting of 28382 complexes and it is disjoint from BINDTEST. Proteins with large sequence identity with the training ones were removed from BINDTEST and BANK.

Figure 1 provides a graphical visualization of the relationships between the diverse datasets that we considered. Furthermore, all protein structures were initially preprocessed using the Schrödinger Protein Preparation Wizard (Schrödinger, The Schrödinger Software. 2020). Exclusively for complexes coming from PDBbind (in particular those of BINDTEST), only those protein chains within a certain distance from the ligand were kept to avoid repetitions of the true pocket. This choice will be better detailed in section “Metrics of interest”.

### The GENEOnet model

**Fig. 2:**
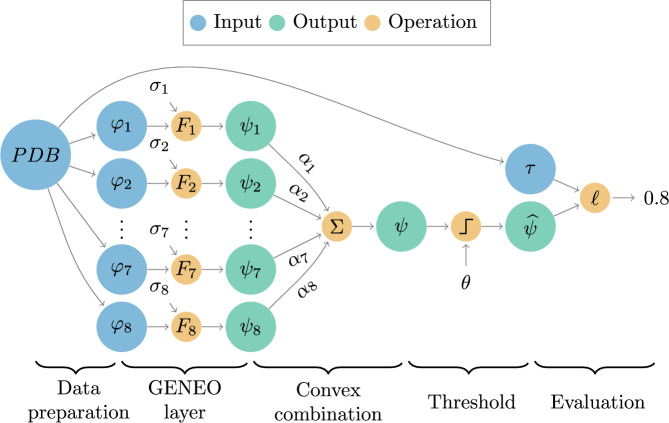
Model workflow. The channels $\varphi _1,\dots ,\varphi _8$, computed from the PDB input file, are fed GENEOs $F_1,\dots ,F_8$ that depend on the shape parameters $\sigma _1,\dots ,\sigma _8$, this first layer returns the intermediate outputs $\psi _1,\dots ,\psi _8$. These outputs are combined through convex combination with weights $\alpha _1,\dots , \alpha _8$ to get the final result $\psi$. To obtain pockets, a thresholding operation with parameter $\theta$ is applied to $\psi$, producing the binary function $\widehat{\psi }$, which finally is compared to the ground truth $\tau$ through the loss function.

GENEOnet, whose architecture is depicted in Fig. 2, consists of five steps: a data preparation step, in which the input data are represented as functions defined on a grid of voxels; a GENEO layer, composed by $d=8$ operators which provides a first processing of the input data; a convex combination of the outputs of the GENEO layer, to obtain new GENEOs which provide a second and more refined processing; a thresholding step which gives a spatial prediction of presence or absence of a pocket; an evaluation step of a loss function which compares the output with the ground truth. The loss function is then optimized, using a training set, to identify the unknown parameters. A further step, applied after the training of the model, allows to compute the scoring of the identified pockets, and consequently their ranking. All the steps are described in detail in the following sections.

#### Data preparation

In the data preparation phase, we represent the protein-ligand complex stored in a PDB file as functions defined in the empty space surrounding the protein, which is discretized using a grid of cubic voxels. For each voxel, we compute approximations of the input functions, or channels $\varphi _i$. As shown in Table 1, such functions reflect a reasoned selection of geometrical, physical and chemical protein properties that are considered to be relevant for pockets detection by medicinal chemistry experts. Auxiliary software called GENEOprep (see Supplementary Information Section 1) was developed to automate the process of computing such channels. The co-crystallized ligand of a protein will be used in the evaluation step to define the true pocket (i.e. the ground truth function $\tau$) for the parameters identification.

**Table 1:** List of potentials that have been used to build GENEOnet.

Name	Type	Expression	Notes
Distance	Geometrical	$\varphi _1(x) = d(x,x_{a^*}) - r_{a^*}$	$x_{a^*}$ and $r_{a^*}$ are coordinates and radius of the nearest atom to the point *x*.
Gravitational	Geometrical	$\varphi _2(x) = \sum _{a\in A} \frac{m(a)}{d(x,x_a)}$	*m*(*a*) is the mass of atom *a*.
Electrostatic	Physical	$\varphi _3(x) = \sum _{a\in A} \frac{q(a)}{d(x,x_a)}$	*q*(*a*) is the partial charge of atom *a*.
Lipophilic	Chemical	$\varphi _4(x) = \sum _{a\in A} \frac{l(a)}{d(x,x_a)}$	*l*(*a*) is the lipophilic coefficient of the atom *a* if it is negative, 0 otherwise.
Hydrophilic	Chemical	$\varphi _5(x) = \sum _{a\in A} \frac{h(a)}{d(x,x_a)}$	*h*(*a*) is the lipophilic coefficient of the atom *a* if it is positive, 0 otherwise.
Polar	Chemical	$\varphi _6(x) = \sum _{a\in A} \frac{p(a)}{d(x,x_a)}$	*p*(*a*) is 1 if atom is polar, 0 otherwise.
HB Acceptor	Chemical	$\varphi _7(x) = \sum _{a\in A} -\varepsilon _a(R^6-2R^4)$	$R = R_{min}/d(x,x_a) + 0.96$ where $\varepsilon _a$ and $R_{min}$ are parameters of the specific type of atom.
HB Donor	Chemical	$\varphi _8(x) = \sum _{a\in A} -\varepsilon _a(R^6-2R^4)\cos ^2\phi _1\cos ^2\phi _2$	$R = R_{min}/d(x,x_a) + 0.96$ where $\varepsilon _a$ and $R_{min}$ are parameters of the specific type of atom, $\phi _1$ and $\phi _2$ are angles defined by triples of points involved in the bond.

In some cases, constants have been ignored because of the subsequent normalization. A summation over all the atoms of the protein would be computationally unfeasible, but, since many potentials depend on the inverse of the distance from *x*, in our computations we neglected atoms too far apart from *x* thus the sums have been computed only for $a\in \mathcal {N}(x)$, where $\mathcal {N}(x)$ is a suitable neighborhood of *x*.

#### GENEO layer

The channels computed in the grid of voxels are then fed to the layer of *d* GENEOs, $F_1,\dots ,F_d$, one per channel. Each operator $F_i$ is chosen from a specific parametric family, parametrized by a shape parameter $\sigma _i$. These families were designed to reflect the a priori knowledge of the experts of medicinal chemistry about the specific role of the corresponding potentials in the pocket identification. We opted for convolutional operators $F_i(\varphi ) = \varphi *K_i$, where $K_i$ are normalized kernels in $L^1(\mathbb {R}^3)$, symmetric with respect to the origin. This choice ensures that all the operators under consideration are indeed non-expansive and equivariant with respect to translations and rotations of the space. We set the parameters regulating the shape of each kernel, so that, also because of their central symmetry, each convolutional operator depends on a single real parameter $\sigma _i$ only, which regulates the “amplitude” of the kernel itself. For the details about the specific kernels employed, refer to the Supplementary Information, Section 2.

#### Convex combination

In the fourth step the intermediate GENEO outputs $\psi _i$ are combined through a convex combination, with weights $\alpha _1, \dots , \alpha _d$ in order to obtain a composite operator $F(\cdot ) = \sum _{i=1}^d \alpha _i F_i(\cdot )$, which is a new GENEO for each choice of the parameters. The output of the convex combination is then normalized to obtain the function $\psi$, defined from $\mathbb {R}^3$ to [0,1]. Here $\psi (x)$ can be read as the likelihood that voxel *x* belongs to a pocket. The coefficients $\alpha _1, \dots , \alpha _d$ can be regarded as feature importance scores, highlighting the importance of each channel in the pocket identification and thus providing a useful tool to explain the results the model delivers.

#### Thresholding

Finally, given a threshold $\theta \in [0,1]$, we get the different pockets returned by the model by taking connected components of the set of voxels where $\psi$ is above $\theta$. In this way, voxels located inside a pocket are labeled with the sequential number of the connected component they belong to, while they are labeled with 0 if they are not judged to belong to any pocket. To summarize, the model that was described so far has a total of 17 learnable parameters ($\sigma _1, \dots , \sigma _d$, $\alpha _1, \dots , \alpha _d$ and $\theta$).

#### Evaluation

For each crystallized complex, the ligand has been converted to the binary function $\tau$ that is equal to 1 on the voxels that (possibly partially) overlap with the ligand, and equal to 0 elsewhere. If we call $\widehat{\psi }$ the output of the model after thresholding, then we have to compare it to the ground truth represented by the binary function $\tau$ in order to asses the goodness of the prediction.

### Training

In order to learn GENEOnet’s learnable parameters, we choose to optimize a loss function evaluating the volumetric matching of ground-truth $\tau$ and prediction $\widehat{\psi }$. The loss function, which needs to be maximized, is defined below:

1 $$\begin{aligned} L(\widehat{\psi }, \tau ) = \frac{|\widehat{\psi } \wedge \tau | + k|(\textbf{1} -\widehat{\psi }) \wedge (\textbf{1} -\tau )|}{|\tau | + k|\textbf{1} - \tau |} \in [0,1] \end{aligned}$$

Here $|\cdot |$ denotes the discretized volume, that is the number of voxels labelled with 1 inside the region, $\widehat{\psi } \wedge \tau$ is a function equal to 1 on the intersection between the prediction $\widehat{\psi }$ and the true pocket $\tau$, $\textbf{1}$ is a constant function equal to 1. All these functions are defined on the voxelized grid built around the protein. The hyperparameter *k* ranges in [0, 1]. We found that values in the range [0.01, 0.05] produce similar results, all characterized by a relatively small number of pockets of appropriate size (see the Supplementary Information, Section 3). Essentially, the choice of the loss function mitigates the imbalance in the number of voxels labeled as part of the pocket in the ground truth. The optimization of $L(\widehat{\psi }, \tau )$ was performed using Adam optimizer. Random sets of 200 proteins uniformly sampled from BIND were used as training sets. We chose this size since empirical evidence showed that increasing the size of the training set did not significantly impact parameter estimates (see the Supplementary Information, Section 6).

### Pocket scoring

In medicinal chemistry, identifying and prioritizing potential ligand-binding pockets is pivotal. This process, essential for streamlining virtual screening, involves evaluating pockets based on their potential to accommodate ligands. Although GENEOnet, as described till now, is able to detect pockets, it does not prioritize them. We can refine this by deriving scores from GENEOnet’s pre-thresholding output. These scores are calculated by averaging the function $\psi (x)$ across voxels within each pocket and adjusting for pocket volume to prevent bias towards smaller pockets. This scoring yields a prioritized set of pockets, facilitating focused and efficient subsequent analyses.

### Metrics of interest

To compare and select different models, we are interested in metrics that express the ability of a model to assign the highest score to the pocket that matches the true one. First of all, we say that a predicted pocket matches the true pocket if it has the largest overlap with the ground truth. By overlap, we mean the ratio between the discretized volume of the intersection and the volume of the true pocket. If no predicted pocket has an intersection with the true one, we say that the method failed on that protein. In this manner, when provided with a dataset of proteins, we can calculate a sequence of coefficients $H_{n + j}$ for $j \ge 0$. Fixing *n* as the protein-dependent number of true pockets, $H_n$ is the fraction of proteins whose ligand is identified within the first *n*-th predicted pockets. On the other hand, $H_{n + j}$ for $j \ge 1$ are the proportions of proteins whose ligand is identified by the $(n + j)$-th predicted pocket. Moreover, we can also consider cumulative sums of these proportions, we generate another sequence of coefficients $T_{n + j}$ for $j \ge 0$. We have that $T_n = H_n$ while if $j \ge 1$ then $T_{n + j} = \sum _{i = 0}^{j} H_{n+i}$ represents the fraction of proteins whose true pocket has been successfully recognized within the first $(n+j)$-th predicted pockets. See the Supplementary Information, Section 4, for the formal definitions of the $H_{n+j}$ and $T_{n+j}$ coefficients. In this way, different methods can be compared as follows: if a method shows higher $T_{n}$ for all $j \ge 0$ then it is the best method. In an optimal situation, we would like to have a model with $H_n = 1.0$ and $H_{n+j} = 0.0$ for every $j \ge 1$. We want to remark that in the case each protein has exactly one true pocket (as in the case of BINDTEST due to the applied preprocessing), we can safely consider $H_1, H_2, \dots$, since $n = 1$ for every protein.

We also consider additional metrics that measure the distance between the predicted pocket centroid and the ground truth. In particular, DCA/PPC is the minimal distance between the ground truth and the centroid of the prediction; on the other hand, DCC is the distance between the centroid of the ground truth and that of the prediction. In literature^24^, usually predictions with DCA/DCC values lower than 4Å are considered successful, and the fractions of successes at different thresholds are usually plotted. Although reporting the results for such metrics, we notice that, differently from the overlap-related ones, DCA and, more importantly, DCC do not take into account the shapes of the ground truth (which may be smaller than the actual pocket) and the predictions. Thus, they shouldn’t be trusted too much in the presence of non-convex pockets or pockets much larger than the ligand; they are well suited to evaluate small and ellipsoid-like pockets.

### Model selection

To assess the reliability of the estimation process and determine the most accurate model for ranking pockets, the loss function *L* was optimized 200 times with different TRAIN sets, all of size 200, each time starting from the same initial guess for the model parameters. For each trained model, BINDVAL was used to calculate the $H_1, H_2, \dots$ coefficients; such results are reported in Supplementary Information, Section 7. Finally, the model having the highest $H_1$ coefficient on BINDVAL was chosen for deployment. The TRAIN set corresponding to such a model, as already stated in section “Data sources”, has a small intersection of size 48 with BINDVAL. We consider this intersection to be negligible, due to the relative size compared to BINDVAL and the fact that BINDVAL is only used to select the optimal version of GENEOnet from the 200 trained instances, not for comparison with other models. The chosen version of TRAIN will also be used in the following in the ablation study described in section “Ablation study”. Moreover, the optimal parameter values of the selected model and additional details about their interpretation can be found in Supplementary Information, Section 5.

### Software and hardware details

The implementation of GENEOnet was achieved through the integration of two distinct software components. The first component comprises a C library, dedicated to computing the potential functions listed in Table 1, while the second module is written in Python. The Python code initiates the computation process by invoking the GENEOprep auxiliary software and the C library via a Cython extension, which enables the calculation of protein potentials. Subsequently, the resulting potentials are passed as input into the GENEOnet network, whose architecture was developed using PyTorch. In terms of computational efficiency, we report that running the optimization algorithm for 50 epochs on the TRAIN dataset results in a processing time of approximately six minutes when performed on a laptop with an NVIDIA GeForce RTX 3060 GPU. In contrast, the same task takes approximately 40 minutes to complete using only the CPU on the same laptop having an 8-core Intel^®^ Core^TM^ i7-10870H processor.

## Results

This Section features the outcome of the experiments designed to evaluate the performance of GENEOnet, including benchmarking against the following state-of-the-art methods that could be accessed by us: Fpocket, P2Rank, DeepPocket, CAVIAR, CavVis.

### Equivariance and non-expansivity combined effect

**Fig. 3:**
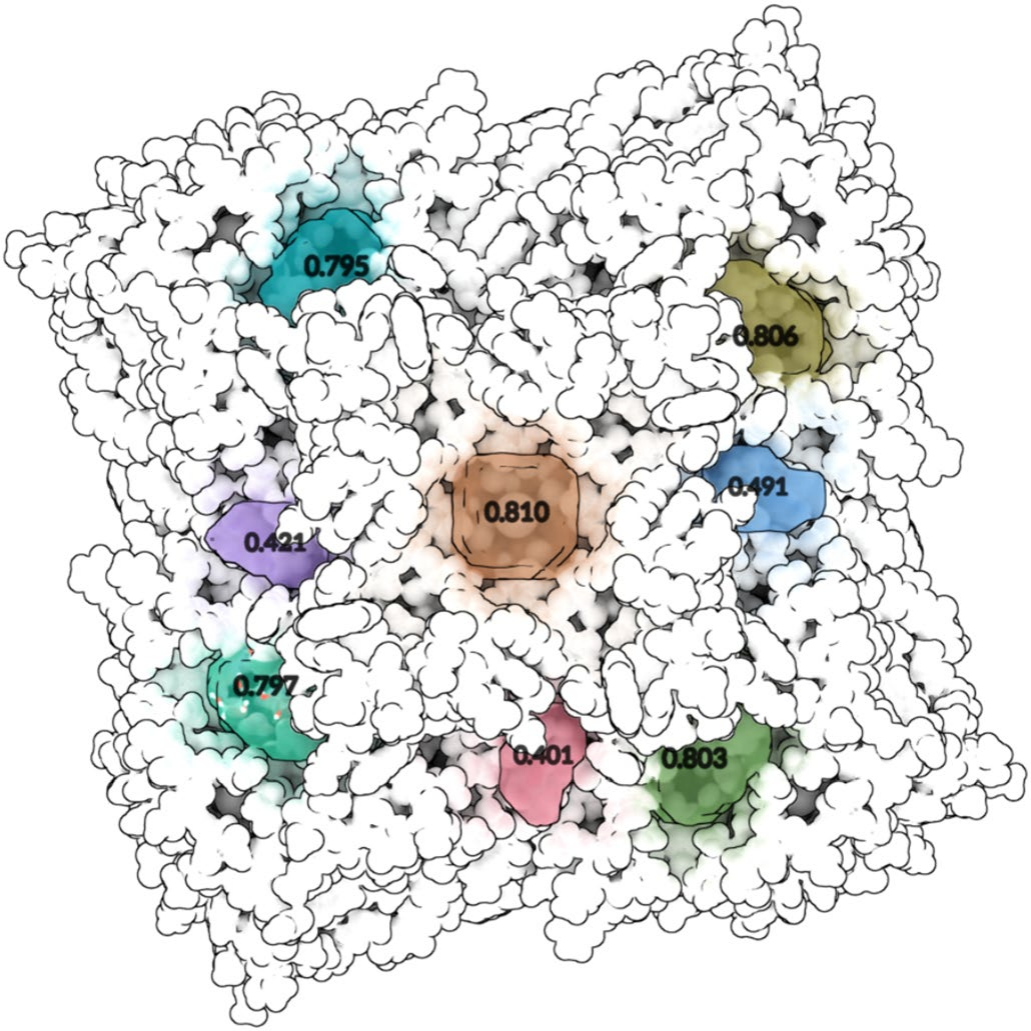
Global view of the prediction: each predicted pocket is shown in a different color and is labeled with its calculated score. In the lower left-hand region is the pocket that correctly identifies the true pocket in which the ligand is located. The ligand is slightly illuminated for better visualization.

Figure 3 shows GENEOnet output for protein (PDB ID 2QWE). This protein has four symmetrical units, resulting in four replicas of the true pocket thus we have $n = 4$. GENEOnet correctly identifies these symmetrical pockets and assigns them high scores (third to sixth top ranked. The fifth predicted pocket matches the ground truth, thus this would contribute to $H_{n + 1}$). This is because results on similar, but differently oriented units, are guaranteed to be similar by the combined actions of equivariance and non-expansivity, moreover, we could expect such a result actually even before running the algorithm. This example highlights the positive effects of equivariance and non-expansivity, effects that are extremely beneficial in determining the robustness and trustworthiness of GENEOnet as already studied in^41,42^. The ablation study of section “Ablation study”, instead, will focus on removing such properties in order to evaluate their impact on model training.

### Pocket identification and ranking

**Fig. 4:**
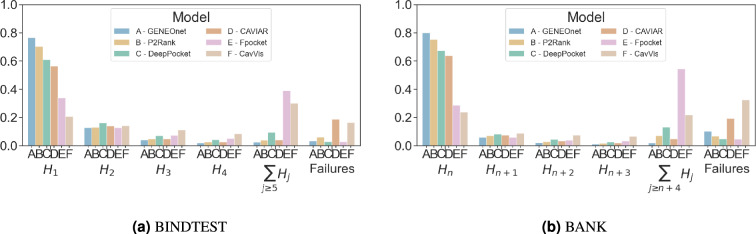
$H_{n+j}$ coefficients. Bar chart of the $H_{n+j}$ coefficients computed on (**a**) BINDTEST and (**b**) BANK for the different methods. On both datasets GENEOnet has the highest value of $H_n$.

The goal of this experiment is to compare different methods in their ability to accurately identify pockets that match the true one and to assign them high scores. Firstly, we report estimates of $H_{n+j}$ coefficients computed on BINDTEST in Fig. 4a. Secondly, the experiment was repeated on BANK and the results are shown in Fig. 4b. Numerical estimates of the coefficients can be found in Supplementary Information, Section 10.

### Overlap analysis

**Fig. 5:**
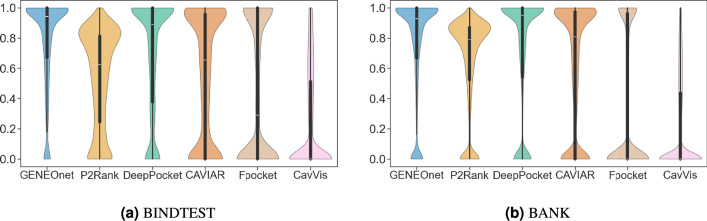
Violin plots of the distributions of the overlaps between the ground truth and the predicted pockets within the top-ranked. The peak of the estimated density in correspondence of 0 highlights the number of failures within the $n+2$ predicted pockets.

After testing the scoring capabilities of the methods, we also compared the ability to identify and to rank on top, pockets that match the true one with high overlap. We computed the distributions of overlaps between the true pocket and the $n + 2$ top ranked pockets for the compared methods (if the method does not hit the true pocket within the $n + 2$ top ranked ones, the overlap is set to 0.0), again first for BIND and then for BANK datasets. Figure 5 shows the distributions of the overlaps using violin plots. The peak of the estimated density in correspondence with zero is related to the proportion of failures within the $n + 2$ predicted pockets. The box plots inside the violins allow for the comparison of the quartiles of the overlap distribution as well.

### Distance metrics

**Fig. 6:**
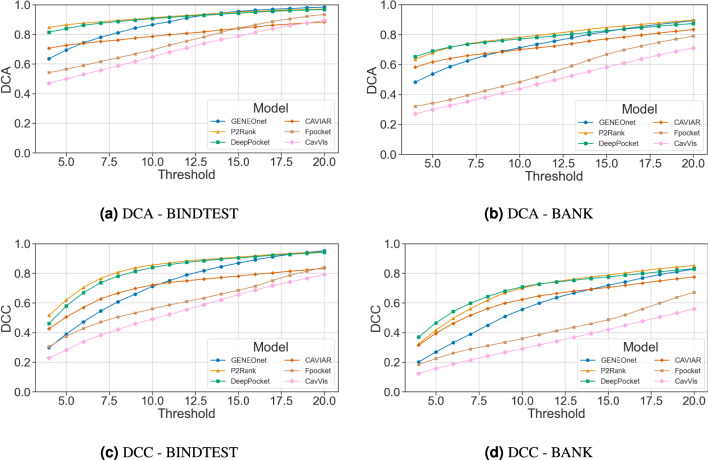
DCA and DCC curves. For each threshold value on the horizontal axis, the proportion of proteins with a DCA/DCC value (the best among the three top-ranked pockets) lower than the threshold is plotted. For the two metrics, the desired model is the one that exhibits the steepest ascent, showing larger success proportions at lower thresholds.

We present here the results of our analysis, which detail the percentages of proteins exhibiting DCA/DCC values below a range of thresholds extending from 4Å to 20Å as computed using both BINDTEST and BANK datasets. The reported DCA and DCC values are the minimum among the three top-ranked pockets for each method, consistent with the overlap analysis presented in the preceding section. Figure 6a–b show the curves for DCA, while Fig. 6c–d show those for DCC.

### Ablation study

To further prove the key importance of equivariance and non-expansivity, we designed an ablation study to compare GENEOnet with other models sharing similar architectures but missing either one or both of the two properties. We will consider three models:

1. An equivariant and non-expansive model (GENEOnet, here E-NE for short).
2. A non-equivariant and non-expansive model (NE-NE for short).
3. A non-equivariant and potentially expansive model (NE-E for short).

For the NE-NE and NE-E models, we replaced the convolutional operators with rotationally invariant kernels of GENEOnet with general convolutional operators, featuring fully learnable kernels, identical in size to those used by GENEOnet. Additionally, for model NE-NE, we normalized the kernels using the $L^1(\mathbb {R}^3)$ norm, consistently with the approach employed by GENEOnet. All three models were trained using the TRAIN set chosen for GENEOnet, with initial parameter and hyperparameter values maintained at their original settings. For this ablation study, an additional test set was generated through sampling 200 proteins with sequence identity less than 80% relative to those in the TRAIN set. This test set was exclusively employed to evaluate potential overfitting of the models.

**Fig. 7:**
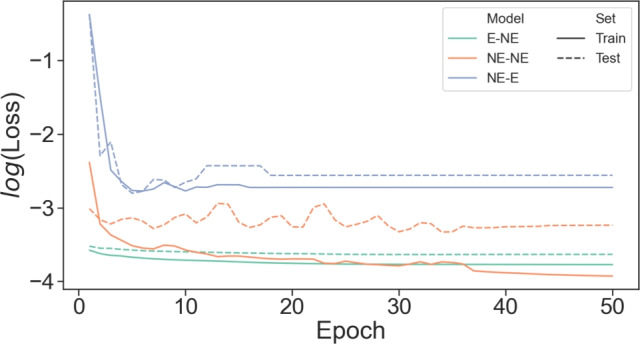
Ablation study: The training and test loss curves are plotted for the three models in the ablation study. Model NE-E is not really able to minimize the loss and learn effectively; model NE-NE learns well on the training set, but it clearly overfits, while model E-NE (GENEOnet) is the only one able to learn effectively while preventing overfitting.

Figure 7 illustrates the evolution of the logarithm of the loss function (1) during training epochs for each model, as indicated by continuous lines representing training loss and dashed lines representing test loss. Notably, model NE-E failed to learn effectively, minimizing the loss, while model NE-NE outperformed GENEOnet in terms of training results but was unable to avoid overfitting. Conversely, model E-NE (GENEOnet) demonstrated the capacity for learning effectively while preventing overfitting of the training data. These findings provide further evidence supporting the importance of equivariance and non-expansivity in achieving an accurate yet parsimonious model. Supplementary Information Section 9 presents additional figures comparing the predictions of each model on one training and one test example.

### Computational time evaluation

To provide a more comprehensive evaluation of GENEOnet’s performance, an additional experiment was conducted to compare the computational costs of the models. Noting that grid-based models, such as GENEOnet, may incur higher computational overhead and larger memory requirements compared to models that exploit only the protein structure, we sought to investigate this aspect further. To do so, we followed this protocol: for each of the 10 protein size classes $(\mathcal {C}_j)_{j=1}^{10}$, where $\mathcal {C}_j$ comprises proteins with a number of atoms ranging from 1000*j* to $1000(j+1)$, we selected the first 100 representative proteins in BIND. Subsequently, for each protein, we ran the methods, this time focusing solely on the computational times.

**Fig. 8:**
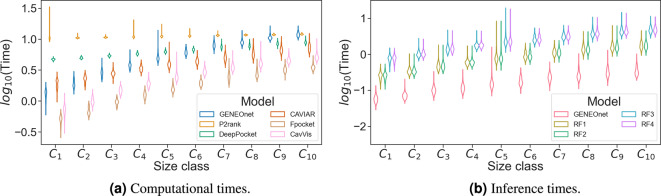
Analysis of computational times. Panel (**a**) shows the distributions of the base-10 logarithm of the total computational times for the considered methods and for the different protein size classes. Panel (**b**) shows a comparison of inference times, once the protein potentials are computed, for GENEOnet and four Random Forest models trained to use GENEOnet potentials.

Figure 8a depicts the estimated distributions of the computational times for the different methods: as foreseeable, Fpocket is the fastest method while CavVis is the second fastest, among the ML approaches; instead, GENEOnet is the fastest method (par with CAVIAR) up to class $\mathcal {C}_5$. For larger proteins, DeepPocket and CAVIAR become faster but are still comparable to GENEOnet in terms of order of magnitude. Regarding P2Rank, the computational times are almost constant with respect to the protein size; this is likely due to the long P2Rank initialization process, while the actual inference is considerably faster. Anyway, among the considered methods, DeepPocket is the one closest to GENEOnet in terms of mechanism and architecture; thus, this analysis shows that GENEOnet speed is better or comparable to DeepPocket on the considered sample. Furthermore, we acknowledge that for GENEOnet, the phase having the highest computational load is the phase of computation of the potentials of Table 1. Although we developed a dedicated C library for this task, the cost of computing the potentials constitutes the large majority of GENEOnet’s computational time. Once the potentials are computed, the inference part of the network is quite fast, as evidenced by Fig. 8b. The plot shows the estimated distributions of the inference computational times for GENEOnet and four Random Forest models that use the same potentials of GENEOnet to generate a prediction for each voxel. The inference time of GENEOnet is lower than all the considered Random Forests. Additional details regarding the models and why GENEOnet’s inference cannot be replaced by a traditional ML approach like Random Forest are provided in n Information Section 9.

### Structural analysis of ABL1 Kinase using GENEOnet

**Fig. 9:**
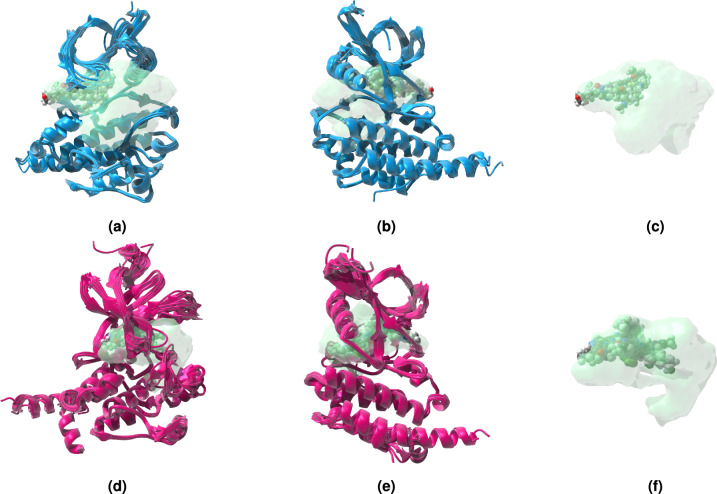
GENEOnet predictions for ABL1 kinase. (**a**,**d**) front and (**b**,**e**) back view of ABL1 aligned structures in the active and inactive conformations mapped with GENEOnet. (**c**–**f**) aligned pockets predicted by GENEOnet.

The performance of GENEOnet was evaluated on a case study concerning multiple X-ray structures of ABL1, both from human and mouse models, in their active and inactive conformations. For the active conformation, only complexes with Type 1 ligands were considered. The structures were aligned and mapped. This case study aimed to compare the pocket identified by GENEOnet with the experimental one, obtained as the space occupied by each ligand in a given structure. As shown in Fig. 9, GENEOnet exhibited excellent results in pocket prediction, capturing all the space occupied by experimental ligands. Moreover, in the active conformation (Fig. 9c), we observed that Type 1 inhibitors and ATP occupy only a portion of the super pocket predicted by GENEOnet. Notably, there is another unexplored region within this binding site that could be useful for enhancing ligand selectivity. Furthermore, in the inactive conformation, the predicted pocket included all experimental ligands, reinforcing the accuracy of GENEOnet in binding pocket prediction.

### GENEOnet, TankBind and AF2BIND comparison on ABL1 Kinase

**Fig. 10:**
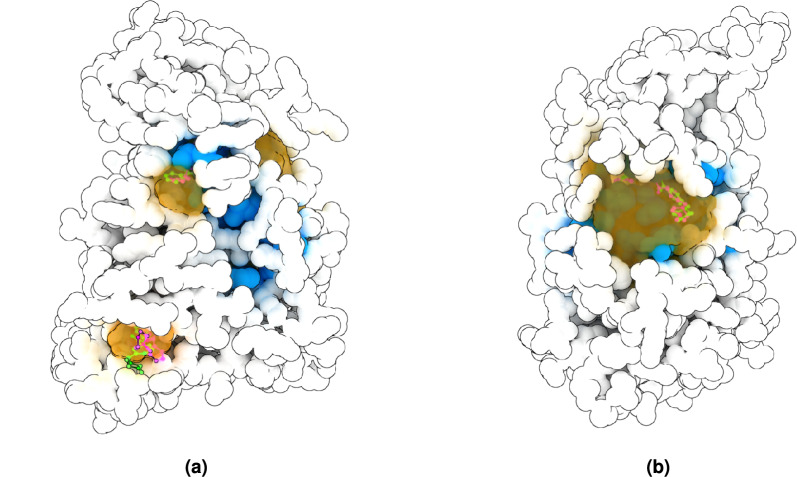
Comparative analysis of GENEOnet, TankBind, and AF2BIND on the protein PDB ID 6HD6. Specifically panels (**a**) and (**b**) display the following elements: experimental ligands STI (located in the middle) and FYH (situated at the lower left), which are depicted in green; TankBind-placed ligands, shown in magenta; residues with a predicted probability of binding **P**(bind) greater than 0.5, as determined by AF2BIND, represented in blue; and GENEOnet’s first two predicted pockets (orange clouds), which scored 0.863 (middle) and 0.690 (lower left), respectively.

We have chosen to compare GENEOnet qualitatively with two other state-of-the-art deep learning and generative AI methods: TankBind^43^ and AF2BIND^44^. However, neither of these methods was designed to produce volumetric predictions, making a comprehensive comparison akin to those presented in previous sections impractical. TankBind is a method that predicts the conformation of a protein-ligand complex given the two structures, utilizing a Graph Neural Network (GNN) architecture to assess the binding affinity of the ligand within specific functional blocks extracted via P2Rank. As such, TankBind is not a ligand-agnostic approach and was initially compared to docking algorithms rather than pocket finders. Nevertheless, we deemed it worthy of qualitative comparison with GENEOnet due to its use of P2Rank, a method previously evaluated in our benchmarks, for prioritizing areas of the protein to be processed. AF2BIND, on the other hand, leverages AlphaFold2^45^ pair features to predict the probability that each residue will contact a small-molecule ligand, given a target protein structure. Specifically, it employs a logistic regression model to assign binding probabilities **P**(bind) to individual residues. We selected the protein PDB ID 6HD6, an example of the ABL1 Kinase that was previously discussed in section “Structural analysis of ABL1 Kinase using GENEOnet” of our case study, as a test structure for evaluating GENEOnet, TankBind, and AF2BIND. The sequence identity of this protein is less than 30.5% with any of the proteins in the TRAIN set used to train GENEOnet, whereas we are uncertain whether it may be present in the training sets of the other two methods. Given these considerations, we applied each of the three methods to the 6HD6 structure predicted by AlphaFold2 and present a comparison of their results in Fig. 10.

An examination of Fig. 10 reveals that GENEOnet successfully identifies both pockets containing the ligands, as well as ranking them among the top two predicted sites. This outcome was anticipated for the central pocket relative to STI, given our analysis in section “Structural analysis of ABL1 Kinase using GENEOnet”. Notably, GENEOnet’s performance is confirmed in identifying the pocket hosting FYH. In contrast, TankBind correctly places both ligands near their experimental binding sites; however, it is worth noting that this method relies on access to the ligand structures for its predictions. In contrast, both GENEOnet and AF2BIND operate as ligand-agnostic predictors. AF2BIND generates a probability of ligandability for each protein residue using logistic regression, which is the primary output of this method. Unfortunately, the authors do not provide a procedure for deriving a finite number of predicted pockets from this outcome. This uncertainty could impact drug design, as it requires accurate selection of the optimal druggable binding site. To facilitate a comparison between AF2BIND and GENEOnet, we have highlighted residues with a probability of ligandability **P**(bind) greater than 0.5, a common choice with logistic regression classification. Under these conditions, AF2BIND successfully identifies the STI pocket, albeit with an additional region that is larger than the one predicted by GENEOnet. Conversely, AF2BIND fails to identify the FYH pocket, although it can be detected if the threshold for **P**(bind) is lowered to approximately 0.3. This adjustment, however, results in a further enlargement of the STI pocket. In conclusion, our analysis indicates that TankBind produces the most precise predictions, albeit under the condition that both ligand structures are available. GENEOnet successfully identifies both pockets and ranks them among the top two predicted sites. AF2BIND, while capable of identifying both pockets at a low threshold for **P**(bind), is unable to detect the second pocket relative to FYH when using larger thresholds.

### Webservice

**Fig. 11:**
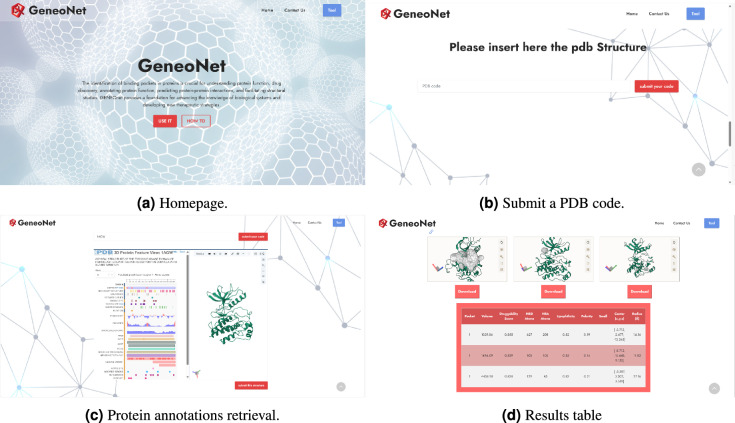
Webservice snapshots.

GENEOnet webservice has been developed to be freely accessible to the scientific community. Figure 11a shows GENEOnet homepage, the “use it” option allows for the submission of the PDB code of the protein of interest (Fig. 11b). By submitting the code, the protein is retrieved from the Protein Data Bank along with every annotation available (Fig. 11c). After submitting the structure, protein pockets identification is performed via GENEOnet and findings are returned in the results table (Fig. 11d). Pockets are described in terms of druggability score, number of hydrogen bond acceptor (HBA) and hydrogen bond donor (HBD) atoms, lipophilicity, and polarity. Measures of the radius and pocket center are also provided. The “small” flag in results table shown in Fig. 11d is marked with an “X” if the pocket is considered small by GENEOnet.

## Discussion and conclusions

In terms of pocket identification accuracy, GENEOnet outperforms all other methods evaluated in the comparison on both the BINDTEST and BANK datasets. Notably, when considering the $T_3$ coefficient on BINDTEST, our results indicate that GENEOnet achieves a value of 0.929, implying that in approximately 93% of cases, the correct pocket can be identified by selecting the top three ranked pockets. All other methods, instead, reach values of $T_3$ below 0.9. Similarly, for BANK, GENEOnet has the highest value of $T_{n+2}$ equal to 0.875. GENEOnet shows a higher number of failures on BANK than BINDTEST, however this should not be considered a problem firstly because all methods have very low failure rates, secondly it is essential to acknowledge that GENEOnet’s final model was chosen to maximise $H_1$ on BINDVAL, in fact, as seen in Fig. 5, when considering only the $n+2$ pockets ranked first, GENEOnet again outperforms the other methods by having the lowest number of failures. On the other hand, DeepPocket/Fpocket, which exhibit the overall lowest failure rate on both datasets, have significantly high failure rates when considering top-ranked pockets. Therefore, in the context of favoring recognitions within the top-ranked pockets, GENEOnet remains the best-performing method, even when considering failures. Regarding the overlap analysis, the experimental results in Fig. 5 show that GENEOnet has one of the most skewed distributions, favouring high overlaps. Additionally, as expected, GENEOnet’s distribution has the smallest number of zero overlap cases (in comparison to DeepPocket/Fpocket, which have similarly skewed distributions). The evaluation of DCA and DCC distances using GENEOnet reveals that it does not outperform other methods, such as P2Rank, DeepPocket, and CAVIAR. However, when considering DCA specifically, GENEOnet’s performance improves significantly and quickly with increasing success thresholds. In contrast, the growth in performance for DCC is slower, and the other methods are only surpassed at larger thresholds. Taking these results into account, we make two key observations. Firstly, it appears that DCA is somewhat insensitive to the ligand’s shape, as its calculation involves determining the minimal distance from the ligand to the centroid of the prediction. In contrast, DCC does not consider either the ligand or pocket shapes, merely computing the distance between the ligand centroid and the centroid of the prediction. Consequently, this metric may be less suitable for evaluating non-convex or larger pockets where the ligand occupies only a portion of the predicted space. From a molecular docking perspective, having slightly larger pockets relative to smaller ones with respect to the ligand is generally desirable. Therefore, while metrics like DCA and DCC are widely reported in the literature, they may not provide valuable insights into the evaluation of pocket finder algorithms that prioritize volumetric inclusion over strict adherence to ground truth (i.e., the cocrystallized ligand). Secondly, these findings can serve as a starting point for improving GENEOnet by incorporating subcavity identification capabilities, similar to those already implemented in CAVIAR. For example, this could be achieved through stricter criteria for identifying predicted units rather than simply considering connected components, as is currently done. We believe that further investigation into this approach may yield beneficial results and warrant future study. Another potential way forward involves the development of a hybrid approach combining several models in a mixture-of-experts approach. In this research work, the primary objective was to identify and select the optimal model based on different performance metrics through a systematic model selection methodology. Future work could involve exploring the integration of distinct GENEOnet models, each of which has been optimized to evaluate different but complementary aspects of the outcome (ranking, overlap, failures, etc.), thus capitalizing on their respective strengths to achieve better predictions and mitigating their weaknesses. As for the identification of subcavities, this deserves further study. In conclusion, results obtained in all the experiments confirm that GENEOnet is able to find and assign high scores to the most likely pockets for a given protein, also with a high overlap. It performs better than the other state-of-the-art models according to many metrics of interest. In cases where GENEOnet does not excel in terms of specific metrics, there are two possible explanations. Firstly, it may be that further refinements to the method itself are warranted, as evidenced by limitations associated with DCA/DCC results and possible subcavity identification. Alternatively, GENEOnet’s performance is on a par with other methods, such as when evaluating computational efficiency for larger proteins. Beyond solely assessing GENEOnet’s performance, this framework also possesses several additional properties that are relevant to its utility and interpretability. Firstly, GENEOnet can incorporate prior knowledge, for example, regarding the significance of lipophilicity properties in protein structure prediction. Furthermore, due to its equivariance, it exhibits insensitivity to irrelevant geometric factors such as the precise location and orientation of the protein, thereby enhancing its robustness. Additionally, empirical evidence from previous studies ^41,42^ has demonstrated that GENEOnet is resilient to minor conformational changes in protein structure. In conclusion, this framework relies on a relatively small number of learnable parameters (only 17), which can be efficiently identified using minimal training data. In fact, as shown by the ablation study, compared to GENEOnet’s non-equivariant and expansive counterpart, GENEOnet requires less training data to converge. Finally, GENEOnet is an interpretable model by design.

## Supplementary Information

Supplementary Information.

## Data Availability

Protein data are derived from the following resources available in the public domain: PDBbind v2020—http://pdbbind.org.cn/ and RCSB Protein Data Bank—https://www.rcsb.org/ GENEOnet website, developed to ensure the scientific community can freely access the tool, is available at the address: https://geneonet.exscalate.eu. The website is based on LAMP software stack (Linux, Apache, MariaDB, PHP). The front-end interface is built on bootstrap 5, jQuery, and HTML5 doctype.
