# Supplementary material for: GENEOnet: a breakthrough in protein binding pocket detection using group equivariant non-expansive operators
Source: Sci Rep. 2025 Oct 3;15:34597. doi: 10.1038/s41598-025-18132-5 (PMC12494700; doi:10.1038/s41598-025-18132-5)
Supplement: Supplementary file 1 — Supplementary Information. [file 41598_2025_18132_MOESM1_ESM.pdf]

GENEOnet: A Breakthrough in Protein Binding Pocket Detection Using Group Equivariant Non-Expansive Operators.

Supplementary Information

Giovanni Bocchi[1], Patrizio Frosini[2], Alessandra Micheletti[1], Alessandro Pedretti[3], Gianluca Palermo[4], Davide Gadioli[4], Carmen Gratteri[5], Filippo Lunghini[6], Akash Deep Biswas[6], Pieter F.W. Stouten[7], Andrea R. Beccari[6], Anna Fava[6], and Carmine Talarico[6]

[1]Dept. of Environmental Science and Policy, University of Milan, Via Celoria 10, 20133 Milano, Italy

[2]Dept. of Computer Science, University of Pisa, Largo B. Pontecorvo 3, 56127 Pisa, Italy

[3]Dept. of Pharmaceutical Sciences, University of Milan, Via Mangiagalli 25, 20133 Milano, Italy

[4]Dept. of Electronics, Information and Bioengineering, Politecnico di Milano, Via Ponzio 34/5, 20133 Milano, Italy

[5]LIGHT S.c.a.r.l., Via Branze 45, 25123 Brescia, Italy

[6]Dompé Farmaceutici S.p.A., Via Tommaso de Amicis 95, 80123 Napoli, Italy

[7]Stouten Pharma Consultancy BV, Kempenarestraat 47, 2860 Sint-Katelijne-Waver, Belgium

1 Input functions and GENEOprep

The auxiliary piece of software GENEOprep is used to obtain the data for computing the potentials from the input files. It is written in C++, and based on VEGA, a molecular modelling suite of programs developed in our laboratories [1]. In particular GENEOprep employs the following pipeline:

1. Reads the PDB file.

2. Adds hydrogens if they are missing.

3. Assigns atomic charges according to the Gasteiger-Marsili method [2].

4. Assigns the Broto-Moreau lipophilicity parameters [3].

5. Searches for atoms which are H-bond acceptors/donors and assigns the non-bond parameters according to CHARMM 36 force field [4].

6. The atom coordinates and the computed data finally are piped to GENEOnet where are used to compute potentials.

2 Parametric families of operators

Regarding the operators introduced in the GENEOnet model to define the GENEO layer, for every channel, but the Distance and the Electrostatic field, we used Gaussian kernels, which are functions $K_i : \mathbb{R}^3 \to \mathbb{R}$ of the type

$$K_i(x) = K_i(r) = e^{-\frac{r^2}{2\sigma_i^2}} \quad i \notin \{1, 3\}$$

where $r = ||x||$ is the distance of the point x from the origin. Our choice is motivated by the need for the corresponding operators to react to the presence of spatial regions showing channels with uniform sign, entirely negative or entirely positive. For the Distance field, instead, we defined a spherical kernel using the radial function

$$K_1(r) = \frac{1}{2} - \tanh(h(r - \sigma_1)) + \frac{1}{2}\tanh(h(r - (\sigma_1 + \beta)))$$

$K_1(r)$ is a differentiable approximation of the step function

$$\widetilde{K_1}(r) = \mathbb{1}_{\{r \leq \sigma_1\}}(r) - \mathbb{1}_{\{r \leq (\sigma_1 + \beta)\}}(r)$$

where $\mathbb{1}$ is the indicator function. The function $\widetilde{K_1}(r)$ takes value +1 inside a sphere of radius σ_1 centered at the origin, -1 inside the spherical shell from radius σ_1 to $\sigma_1 + \beta$ and 0 outside the sphere of radius $\sigma_1 + \beta$, where β is the thickness of the shell. From the definition of the channel φ_1 in Table 1 of the paper we see that the Distance potential is negative in the interior of the protein surface and positive in the exterior. Thus the negative value inside the spherical shell of the kernel ensures that positive regions far from the protein surface will not provide a high contribution to the convolution. In this way, the operator using this kernel will detect only spherical voids close to the protein surface that are surrounded by protein atoms. The hyperparameter $\beta \in \mathbb{R}_+$ tunes how much we are interested in the region outside the inner sphere: higher values mean that we look for pockets buried deep inside the protein. Finally the hyperparameter $h \in \mathbb{R}_+$ measures to which extent K_1 is a good approximation of $\widetilde{K_1}$, where higher values lead to a better approximation, but to almost non-differentiability, that could be an issue during the optimization step. We fixed $\beta = 1.5$ and $h = 100$ in GENEOnet, since these values empirically gave good results. For the Electrostatic field we used a Gaussian Laplacian kernel

$$K_3(r) = \left(\frac{3}{\sigma_3^2} - \frac{r}{\sigma_3^4}\right) e^{-\frac{r^2}{2\sigma_3^2}}$$

since we are interested in finding suitable variations in the charges of the atoms inside the pocket. Except for the kernel applied to the Distance channel, where the shape parameter σ_1 is the radius of the positive sphere, for all the other kernels the shape parameters are the standard deviations of the Gaussians.

3 The hyperparameter k of the loss function

We observe that by assuming $k = 1$ in the loss function in the Training subsection of Materials and Methods, more importance is given to correctly recognizing the voxels that do not contain the ligand (referred to as the "non-cavity") rather than accurately identifying the voxels where the ligand is present (referred to as the "cavity"). This emphasis on the "non-cavity" is due to the fact that the ligand, and consequently the cavity, usually occupies a much smaller volume compared to the total protein. As a result, assuming $k = 1$ would not direct the method towards the accurate identification of the true pocket. Furthermore, it would hinder the identification of other potential pockets that could serve as suitable locations for hosting other ligands. For these reasons, we opt for $k < 1$, which enables a better balance between the two terms in the numerator. This approach leads to the identification of a slightly bigger number of larger pockets. The opposite extreme occurs when $k = 0$. In this scenario, the model generates pockets that can be as large as the entire grid, because the only important criterion is that each voxel containing the ligand is labeled with 1. Taking this into consideration, selecting a value of k strictly between 0 and 1 yields the best outcomes.

4 Formal definitions of Metrics of Interest H_{n+j} and T_{n+j}

First of all we say that a predicted pocket $A \subset \mathbb{R}^3$ matches the true pocket $B \subset \mathbb{R}^3$ if A has the greatest overlap with B, where the overlap o between two pockets is defined as:

$$o(A, B) = \frac{|A \wedge B|}{|B|} \in [0, 1] \tag{1}$$

In the previous expression, $|\cdot|$ denotes the 3D discretized volume of the region, that is the number of voxels in the region. In the following when writing "A matches B" we mean that A is the predicted pocket that has the highest overlap with B, i.e. $A = \arg\max_C o(C, B)$ where C ranges in the set of predicted pockets. If no predicted pocket has an intersection with the true one, we say that the method failed on that protein.

In the following, we will denote by m the size of the considered dataset of proteins and with n the number of possible true pockets. Now we recall the coefficient $H_n^{\mathcal{M}}$, defined in the Metrics of Interest section, which is the proportion of correct recognitions within the n-th top ranked pocket of a model $\mathcal{M}$:

$$H_n^{\mathcal{M}} = \frac{1}{m} \sum_{t=1}^{m} \max_{j=1}^{n} \{\Delta_j(\mathcal{M}(P_t), \tau_t)\} \tag{2}$$

Supplementary Table 1: Optimal parameters of selected model.

Unit	Channel	σ_i	α_j	θ
1	Distance	3.110	0.362	0.756
2	Gravitational	5.197	0.002	
3	Electrostatic	2.561	0.054	
4	Lipophilic	4.678	0.338	
5	Hydrophilic	3.545	0.001	
6	Polar	6.166	0.185	
7	HB Acceptor	4.186	0.056	
8	HB Donor	3.908	0.001	

Then for every $j \geq 1$ we have:

$$H^{\mathcal{M}}_{n+j} = \frac{1}{m} \sum_{t=1}^{m} \Delta_{n+j}(\mathcal{M}(P_t), \tau_t) \tag{3}$$

In Equations 2 and 3, the symbol $\mathcal{M}(P_t)$ denotes the set of predicted pockets by model $\mathcal{M}$ applied to protein P_t, τ_t is the true pocket of the protein P_t, while the expression $\Delta_j(\mathcal{M}(P_t), \tau_t)$ is defined as

$$\Delta_j(\mathcal{M}(P_t), \tau_t) = \begin{cases} 1 & \text{if } j = \arg\max_{l \geq 1} o(\mathcal{M}(P_t)_l, \tau_t) \\ 0 & \text{Otherwise.} \end{cases}$$

where $\mathcal{M}(P_t)_l$ is the l-th top ranked pocket returned by model $\mathcal{M}$ applied to protein P_t. We may also define the corresponding cumulative quantities that can be used to compare different models, in particular $T^{\mathcal{M}}_n = H^{\mathcal{M}}_n$, then for every $j \geq 1$:

$$T^{\mathcal{M}}_{n+j} = \sum_{i=0}^{j} H^{\mathcal{M}}_{n+j}.$$

These definitions take up in a more formal way those given in the subsection Metrics of Interest of Materials and Methods.

5 Parameters intepretability

Optimal parameters can be given clear and comprehensible meanings that clarify their role in the generation of predictions. For example, the coefficients of the convex combination α_j can be seen as the feature importance of the various channels, thus identifying those most influential in obtaining the prediction. Looking at Supplementary Table 1, the Distance and the Lipophilic channels have the highest importance. Moreover the shape parameters σ_i are directly linked to the shapes of the kernels as illustrated in Section 2.

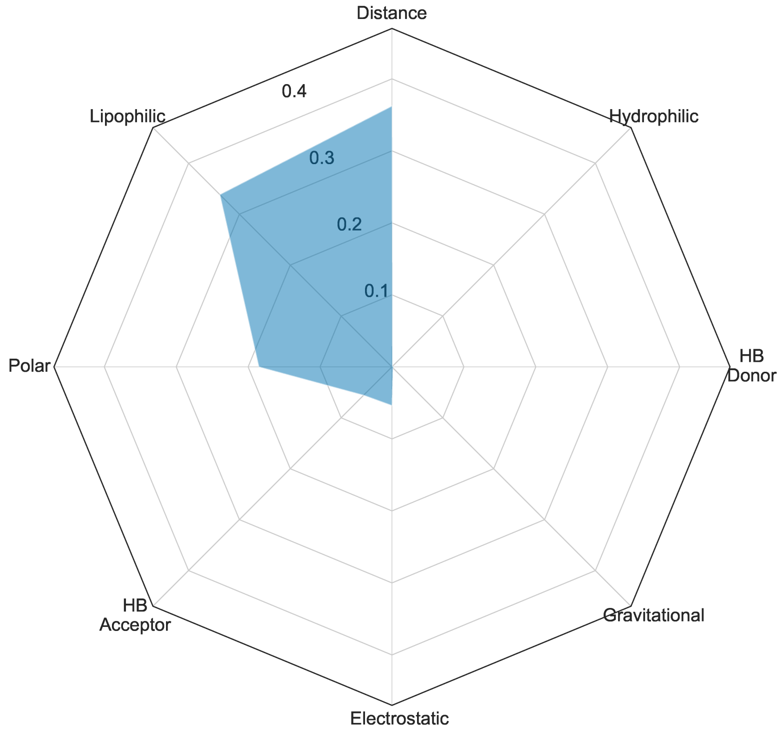

Supplementary Figure 1: Radar plot of the convex combination coefficients $\alpha_1, \ldots, \alpha_d$, ordered anticlockwise with respect to their magnitude.

6 Size of the training set

In order to choose the appropriate size for the training set, we made the following experiment: using BIND, we iteratively trained a family of 31 models starting from a training set of size 5 up to a training set of size 305. At each step, a new model is obtained starting from the same initial guess for the parameters, but with a training set whose size is augmented by 10. The added molecules are randomly sampled in the entire BIND, as well as the starting 5. For each iteration, we recorded the values of the parameters after the optimization, and the results are shown in Supplementary Figure 2.

The plots show a progressive stabilization of most of the parameter values as the size of the training set increases. This behavior could be expected, since the small number of parameters of GENEOnet does not need a huge training set to be identified. Consequently, we chose to adopt a training set of size 200, since from the plots we can see that almost all the parameters have reached a stable value (still accepting a little fluctuation), and this choice proves to be a good compromise between accuracy and training time reduction.

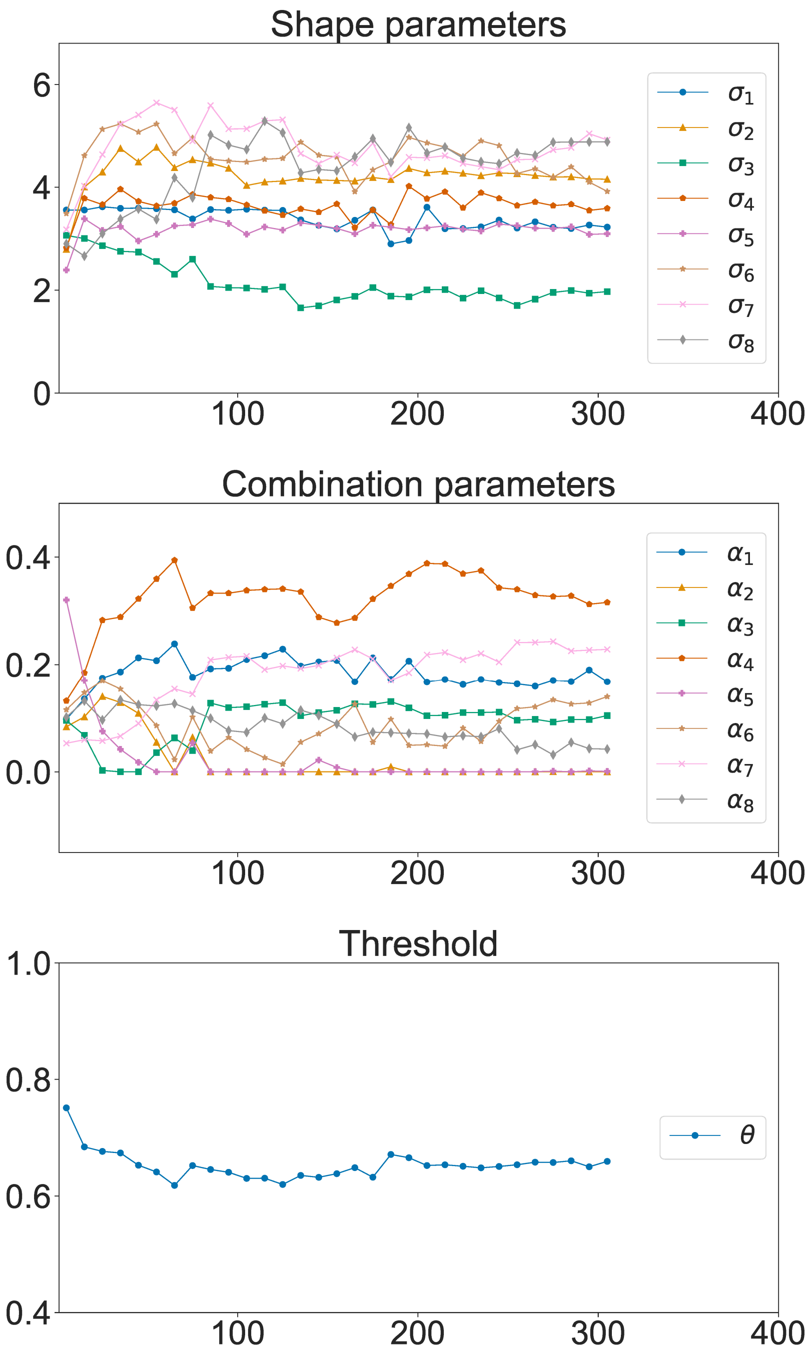

Supplementary Figure 2: This Figure shows the evolution of the values of the optimal parameters as a function of the size of the training set. The upper plot is relative to the shape parameters, the central one to the convex combination parameters, and the lower one to the threshold.

Index	H_1	H_2	H_3	H_4	$\sum_{j\geq 5} H_j$	failures
148	0.805	0.102	0.033	0.014	0.017	0.028
116	0.789	0.107	0.039	0.015	0.024	0.027
18	0.787	0.112	0.036	0.017	0.021	0.028
62	0.786	0.113	0.044	0.014	0.021	0.022
115	0.786	0.113	0.044	0.014	0.021	0.022
88	0.785	0.118	0.038	0.015	0.023	0.021
150	0.782	0.116	0.038	0.013	0.024	0.027
67	0.782	0.116	0.038	0.013	0.024	0.027
181	0.781	0.115	0.044	0.013	0.019	0.029
57	0.781	0.121	0.038	0.015	0.022	0.023

Supplementary Table 2: Performances of the best models on BINDVAL. We report the performances of the ten models with highest H_1 as computed on BINDVAL.

7 Choice of the training set and estimation robustness

Always using BIND, we generated 200 different models, all starting from the same initial values of the parameters but with different training sets of size 200. Each training set was sampled uniformly at random, and it was used to train the corresponding model. Optimal values of the parameters were recorded and used to generate the box plots in Supplementary Figure 3.

The boxplots may help to provide deeper interpretations of the parameters: for example, the overall distribution of α_5, that is the coefficient of the Hydrophilic channel, is very concentrated towards 0 meaning that the corresponding estimated coefficient will rarely be much higher than 0; consequently the Hydrophilic channel will always have a low importance for the final pocket prediction. This, rather than suggesting the inutility of Hydrophilic properties, may suggest that they are better captured by other channels, such as the Polar one. On the contrary, the distributions of α_1 and α_4 are spread around values quite above 0, meaning that the Distance and the Lipophilic channels, of which α_1 and α_4 are the coefficients, respectively, seem to always give a significant contribution to the final prediction.

The 200 trained models are optimal with respect to the loss function defined in Equation (1) of Section 2.3, but this does not guarantee that the models are optimal also with respect to pocket scoring. In order to select the overall best model, we compared them on BINDVAL by computing the coefficients H_{n+j} (in this case equivalent to H_j since each protein was preprocessed to avoid repetitions of the pocket by symmetry) and ordering the models for decreasing values of H_1. As noticed in Section 2.1, we acknowledge that all the sampled TRAIN sets have a small random intersection with BINDVAL: the mean size of this intersection is 52, with a minimum of 35 and a maximum of 64, as we could expect since the training sets are sampled uniformly at random from BIND. Anyway, this fact may be considered negligible, since the intersection is always smaller than the 2% of BINDVAL and BINDVAL was used only for the task of selecting the optimal model (that for this reason could be slightly suboptimal), while BINDTEST was employed in the comparison with other methods. Results are shown in Supplementary Table 2.

Supplementary Table 2 shows on top the model that was chosen as our final version of GENEOnet, and its TRAIN set was considered optimal both for the loss function and for the scoring of the pockets. This model was used to perform the experiments described in the Results section and to deploy the webservice. Finally, we remark that while we admitted

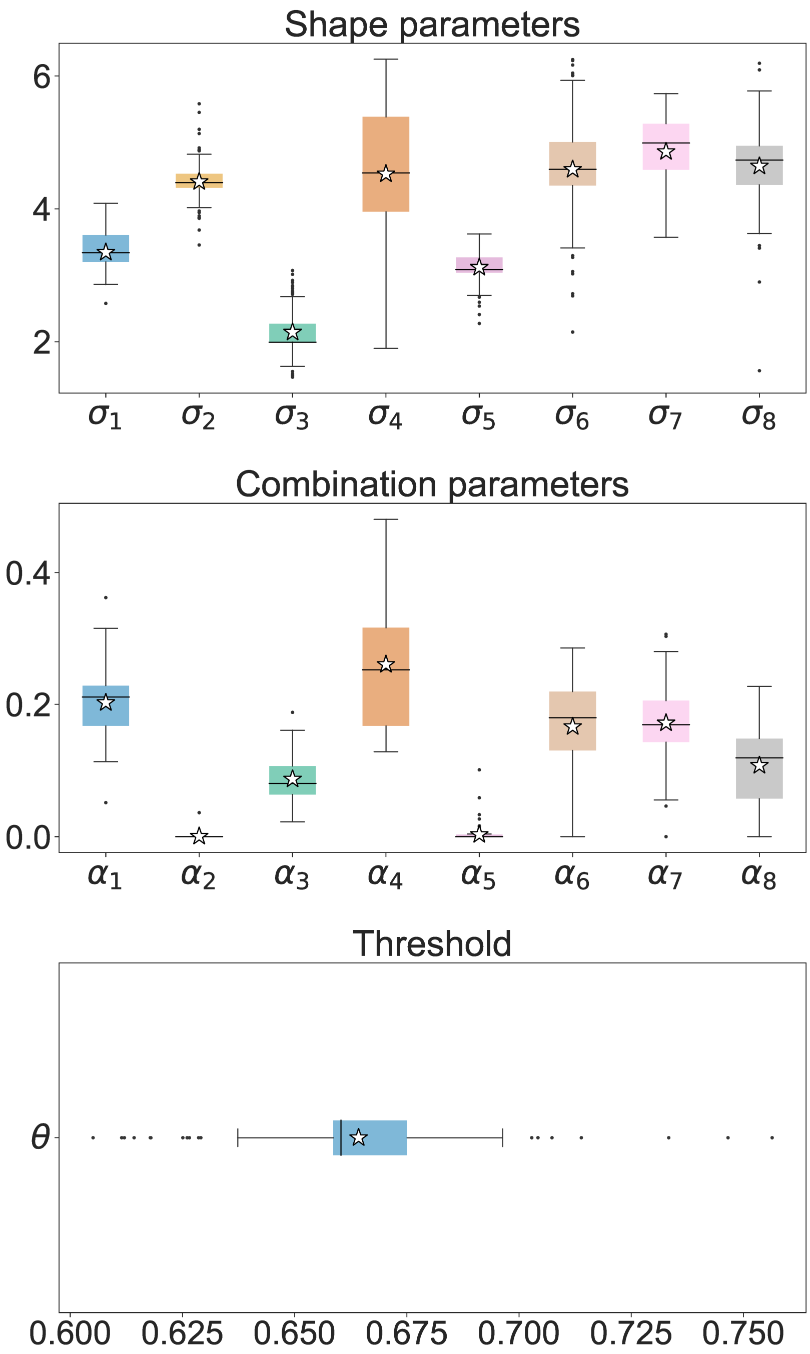

Supplementary Figure 3: These box plots show the distributions of the parameters. In the upper panel, the distributions of the shape parameters are plotted, in the central panel those of the convex combination parameters are reported, and in the lower panel the distribution of the threshold is depicted.

a small intersection between the training set and BINDVAL, we avoided any overlapping between the training set of the best selected model and BINDTEST as described in Section 2.1.

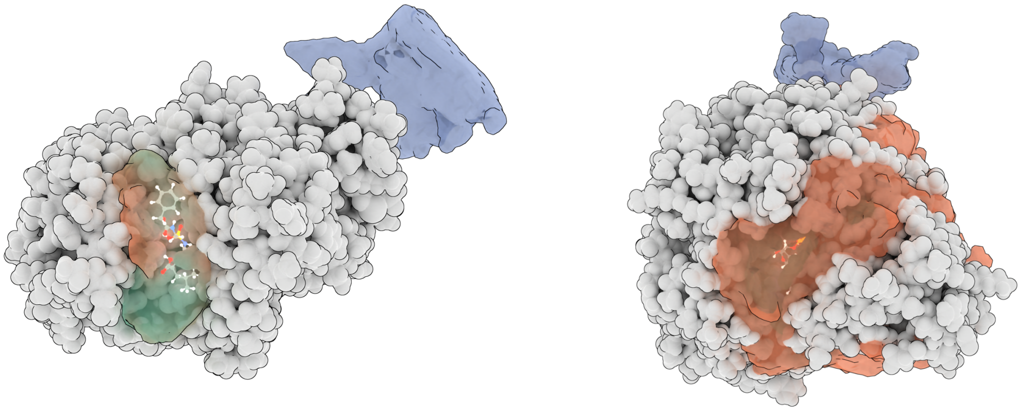

(a) 5M9W (b) 3RUP

Supplementary Figure 4: Comparison of the predictions of the three models of the Ablation Study on a protein from TRAIN and a protein outside TRAIN. Panel (a) protein 5M9W belonging to TRAIN, Panel (b) protein 3RUP in the ablation test set.

8 Ablation study

In this Section, we report a pair of Figures showing the predictions of the three models considered in the ablation study of Section 3.5. We considered a protein, belonging to TRAIN, with PDB ID 5M9W, and a protein belonging to the ablation test set with PDB ID 3RUP. Both the panels of Supplementary Figure 4 show that the NE-E model (blue) provides implausible predictions, Supplementary Figure 4a shows that both GENEOnet (green) and the NE-NE model (orange) provide a good prediction for a protein belonging to TRAIN, while Supplementary Figure 4b shows that the NE-NE model is not able to return a plausible prediction for a protein outside TRAIN, as instead GENEOnet does.

9 Computational times and Random Forests

Here we specify the details of the Random Forest models discussed in Section 3.6. We considered four models imposing different bounds on the maximal depth of each tree in the forest:

1. RF1: maximal depth 2.
2. RF2: maximal depth 5.
3. RF3: maximal depth 20.
4. RF4: maximal depth 100.

Each model was trained to predict, given the values of the eight GENEOnet potentials in a voxel, a binary label which encodes the belonging of that voxel to a pocket. The global prediction is then obtained by running the model for every voxel in the grid. To train the

Random Forests, we subsampled a suitable set of the voxels of the grids computed for the proteins in TRAIN, to guarantee a balanced distribution of the two labels that otherwise would be heavily unbalanced.

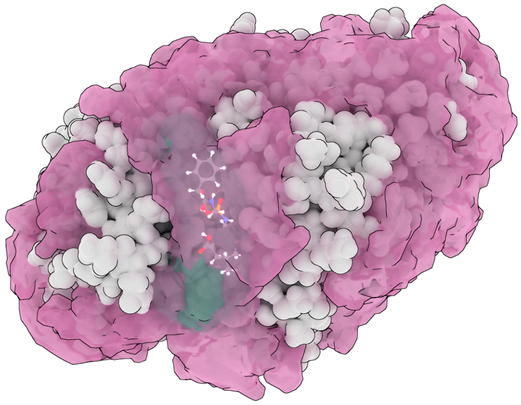

Supplementary Figure 5: Comparison of GENEOnet (green) and RF4 (pink) predictions on protein with PDB ID 5M9W. Even if the voxels of such protein are contained in the training set used to fit RF4, such a model is not able to generate a plausible prediction and suffers from a high number of false positives.

As shown in Supplementary Figure 5, RF4 (as well as the other models based on Random Forests) suffer from a very high number of false positives. Thus, GENEOnet inference network cannot be replaced by Random Forests, neither from the point of view of computational efficiency nor from the point of view of prediction accuracy.

10 Supplementary results

Here we report the Tables containing the numerical values of $H_{n+j}^{\mathcal{M}}$ and $T_{n+j}^{\mathcal{M}}$ for both BINDTEST and BANK. They express the same results shown using bar plots in Figure 4 of the Results section.

Method	H_1	H_2	H_3	H_4	$\sum_{j\geq 5} H_j$	failures
GENEOnet	0.764	0.127	0.039	0.017	0.023	0.031
P2Rank	0.702	0.128	0.048	0.026	0.037	0.059
DeepPocket	0.609	0.160	0.070	0.041	0.093	0.028
CAVIAR	0.563	0.138	0.048	0.025	0.039	0.186
Fpocket	0.337	0.128	0.071	0.049	0.388	0.028
CavVis	0.205	0.140	0.111	0.083	0.299	0.162

Supplementary Table 3: H_{n+j} (i.e. H_j) values for the tested methods on BINDTEST.

Method	T_1	T_2	T_3	T_4	$\sum_{j\geq 1} H_j$
GENEOnet	**0.764**	**0.891**	**0.929**	**0.946**	0.969
P2Rank	0.702	0.830	0.878	0.904	0.941
DeepPocket	0.609	0.769	0.838	0.879	**0.972**
CAVIAR	0.563	0.702	0.749	0.774	0.814
Fpocket	0.337	0.465	0.536	0.584	**0.972**
CavVis	0.205	0.345	0.455	0.539	0.838

Supplementary Table 4: T_{n+j} (i.e. T_j) for the tested methods on BINDTEST. Bold numbers are used to denote, for every coefficient, the method(s) that provided the best result.

Method	H_n	H_{n+1}	H_{n+2}	H_{n+3}	$\sum_{j\geq 4} H_{n+j}$	failures
GENEOnet	0.799	0.056	0.020	0.009	0.017	0.100
P2Rank	0.752	0.069	0.028	0.015	0.069	0.067
DeepPocket	0.672	0.082	0.043	0.026	0.130	0.048
CAVIAR	0.636	0.074	0.032	0.018	0.048	0.193
Fpocket	0.285	0.057	0.038	0.031	0.543	0.046
CavVis	0.235	0.087	0.072	0.065	0.217	0.324

Supplementary Table 5: H_{n+j} values for the tested methods on BANK.

Method	T_n	T_{n+1}	T_{n+2}	T_{n+3}	$\sum_{j\geq 0} H_{n+j}$
GENEOnet	**0.799**	**0.855**	**0.875**	**0.884**	0.900
P2Rank	0.752	0.822	0.850	0.864	0.933
DeepPocket	0.672	0.753	0.796	0.822	0.952
CAVIAR	0.636	0.710	0.742	0.760	0.807
Fpocket	0.285	0.343	0.380	0.411	**0.954**
CavVis	0.235	0.323	0.395	0.460	0.676

Supplementary Table 6: T_{n+j} values for the tested methods on BANK. Bold numbers are used to denote, for every coefficient, the method(s) that provided the best result.

References

[1] A. Pedretti, A. Mazzolari, S. Gervasoni, L. Fumagalli, and G. Vistoli, "The VEGA suite of programs: an versatile platform for cheminformatics and drug design projects," Bioinformatics, vol. 37, no. 8, pp. 1174–1175, May 2021, doi: 10.1093/bioinformatics/btaa774.

[2] J. Gasteiger and M. Marsili, "Iterative partial equalization of orbital electronegativity—a rapid access to atomic charges," Tetrahedron, vol. 36, no. 22, pp. 3219–3228, Jan. 1980, doi: 10.1016/0040-4020(80)80168-2.

[3] P. Broto, G. Moreau, and C. Vandycke, "Molecular structures: perception, autocorrelation descriptor and sar studies: system of atomic contributions for the calculation of the n-octanol/water partition coefficients," European Journal of Medicinal Chemistry, vol. 19, pp. 71–78, 1984.

[4] R. B. Best et al., "Optimization of the Additive CHARMM All-Atom Protein Force Field Targeting Improved Sampling of the Backbone ϕ, ψ and Side-Chain chi(1) and chi(2) Dihedral Angles," Journal of Chemical Theory and Computation, vol. 8, no. 9, pp. 3257–3273, Sep. 2012, doi: 10.1021/ct300400x.

[5] C. C. Ayala-Aguilera, T. Valero, A. Lorente-Macias, D. J. Baillache, S. Croke, & A. Unciti-Broceta, "Small molecule kinase inhibitor drugs (1995–2021): medical indication, pharmacology, and synthesis". Journal of Medicinal Chemistry, 65(2), 1047-1131. DOI: 10.1021/acs.jmedchem.1c00963

[6] M. Okay, I.C. Haznedaroglu, "Protein Kinases in Hematological Disorders. In: Engin, A.B., Engin, A. (eds) Protein Kinase-mediated Decisions Between Life and Death. Advances in Experimental Medicine and Biology", vol 1275. Springer, Cham. DOI: 10.1007/978-3-030-49844-3_15

[7] V. Modi, & R. L. Dunbrack Jr, "Defining a new nomenclature for the structures of active and inactive kinases", Proceedings of the National Academy of Sciences, 116(14), 6818-6827, DOI: 10.1073/pnas.1814279116

[8] Y. Pan, & M. M. Mader, "Principles of kinase allosteric inhibition and pocket validation", Journal of Medicinal Chemistry, 65(7), 5288-5299, DOI: 10.1021/acs.jmedchem.2c00073
